# *EBioMedicine*: Bridging Two Cultures to Improve Health

**DOI:** 10.1016/j.ebiom.2014.10.007

**Published:** 2014-10-22

West Africa is currently in the grip of the worst outbreak of Ebola virus in documented history, eclipsing past outbreaks in terms of both the number of people infected and the number of fatalities. In the 38 years since the discovery of the virus, we still do not have an effective vaccine or antiviral treatment for this devastating pathogen, although at least three vaccines are in development. As with many neglected tropical diseases, Ebola has not historically been considered a major international public health priority. Sentiment is now clearly changing, and the urgency regarding the need for prevention and treatment in response to this virus is now being recognized as cases and virus transmission are being seen in non-African countries.

We are launching *EBioMedicine* as a centralized forum where clinicians and scientists in both the clinic and the laboratory can communicate their observations, ideas, and research insights—and work together to address outbreaks such as Ebola and other major biomedical health challenges. With specific regard to the Ebola crisis we would, for example, consider primary research papers looking at evolutionary mechanisms of pathogenesis or viral drug response and resistance; studies identifying new targetable pathogen vulnerabilities and vaccine strategies; new screening approaches; analyses of disease transmission, progression and recovery; and opinion pieces about why this outbreak is so severe. In this inaugural issue, we are pleased to present an *In Focus* commentary from Kawaoka, Feldmann, and colleagues, who highlight vaccine and treatment challenges facing both basic and clinical researchers working on the current Ebola threat.

However, *EBioMedicine*'s scope is not limited to viral outbreaks. The journal will also cover the entire spectrum of biomedical research—we will consider basic and clinical research papers addressing, but not limited to, the general subjects of cancer, microbiology, immunology, the microbiome, metabolism, obesity, cardiovascular disease, diabetes, neurodegenerative and neurodevelopmental diseases, stroke, allergy, autoimmunity, inflammatory bowel diseases, vaccine design, regenerative medicine, and aging. We will also consider papers discussing technological approaches to expediting and applying research insights such as bioinformatics, imaging, and personalized medicine; -omics research; and whole-genome sequencing studies. We expect our scope to be dynamic and broad, inextricably tied to the evolution and emergence of biomedical health concerns, but always focused on bringing scientists and physicians together to transform information to action in improving human health.

*EBioMedicine* is a translational journal, but how do we define translational research? Technology has become so powerful and so nimble that personalized whole-genome sequencing is likely to be within our reach as a practical clinical tool. For example, clinicians may now use genomic screening to stratify cancer patients into groups that might benefit from targeted, gene-specific treatments that are in turn derived from recent basic science advances in the underlying biology of those altered genes. Translational medicine endeavors to bring basic researchers and clinicians together in a common space where great discoveries in the laboratory can be applied directly and rapidly in the clinic. The field also depends on the input and feedback from those at the forefront of clinical care—their daily observations in the clinic being critical for both detecting the emergence of new public health concerns and communicating the progress of new treatment modalities. Translational research is therefore inherently bidirectional and far reaching in its scope and applications.

We live at a time when a highly pathogenic viral epidemic appears to be spiraling out of control, with no clear resolution in sight. But, we also live in an era with unprecedented resources to combat this threat and others like it. With the launch of *EBioMedicine*, we hope to serve both the clinical and basic research communities by offering a multimedia platform to facilitate dialogue where scientific ideas and clinical needs can be defined, experimentally explored, and ultimately solved.

